# Male subfertility and oxidative stress

**DOI:** 10.1016/j.redox.2021.102071

**Published:** 2021-07-18

**Authors:** Emily P.P. Evans, Jorien T.M. Scholten, Aldona Mzyk, Claudia Reyes-San-Martin, Arturo E. Llumbet, Thamir Hamoh, Eus G.J.M. Arts, Romana Schirhagl, Astrid E.P. Cantineau

**Affiliations:** a Department of Biomedical Engineering, Groningen University University Medical Center Groningen, Antonius Deusinglaan 1, 9713AW, Groningen, the Netherlands; b Institute of Metallurgy and Materials Science, Polish Academy of Sciences, Reymonta 25, 30-059, Krakow, Poland; c Laboratory of Genomic of Germ Cells, Biomedical Sciences Institute, Faculty of Medicine, University of Chile. Independencia, 1027, Independencia Santiago, Chile; d Department of Obstetrics and Gynaecology, University of Groningen, University Medical Center Groningen, Groningen, the Netherlands

**Keywords:** Reactive oxygen species, Oxidative stress, Infertility, Subfertility, Sperm

## Abstract

To date 15% of couples are suffering from infertility with 45–50% of males being responsible. With an increase in paternal age as well as various environmental and lifestyle factors worsening these figures are expected to increase. As the so-called free radical theory of infertility suggests, free radicals or reactive oxygen species (ROS) play an essential role in this process. However, ROS also fulfill important functions for instance in sperm maturation. The aim of this review article is to discuss the role reactive oxygen species play in male fertility and how these are influenced by lifestyle, age or disease. We will further discuss how these ROS are measured and how they can be avoided during in-vitro fertilization.

## Graphical abstract

Here we review how reactive oxygen species are created in sperm cells and how they influence fertility. We include a discussion on physiology and pathophysiology. We further review lifestyle factors and issues related to in-vitro fertilization.

**Figure:**
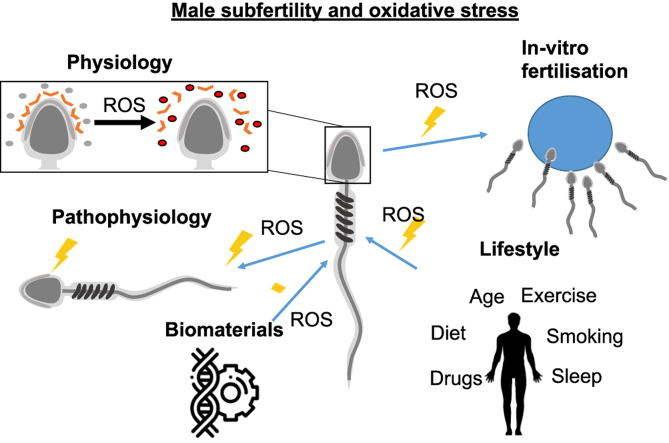

## 1 Introduction

Infertility occurs in approximately 15% of couples [1]. Here, infertility is the inability of a sexually active, non-contracepting couple to achieve pregnancy within one year [2]. Male infertility factors contribute in 45%–50% of these cases, with 7% of all men worldwide diagnosed as infertile [3]. Relatively recent efforts have been made to address the idiopathic causes of male subfertility both clinically and in the laboratories. At cellular level, researchers have underlined the significant contribution of oxidative stress. Oxidative stress is defined as a pathological imbalance between ROS and antioxidants. Specifically, it refers to the excessive production of ROS, overwhelming the eliminatory system of antioxidants [4,5]. ROS are highly reactive oxygen derived molecules with one or more unbound electrons [6]. In the correct balance, ROS play an important role in sperm: They contribute to capacitation, the acrosomal reaction and spermatozoon-oocyte fusion [6,7]. The pathological disbalance can be caused by multiple factors: excessive endogenous or exogenous ROS production, depleted supply of antioxidants, inactivation or diminished production of antioxidant enzymes or a combination of the aforementioned [8]. Examples that may cause abundant production of ROS are infection, varicocele (enlargement of a vein in the scrotum), cigarette smoking, alcohol and drug use, and environmental pollution [5].

ROS-mediated damage to spermatozoa and decreased levels of seminal total antioxidant capacity may be causative factors in 30–80% of infertile men [9,10]. The plasma membrane of spermatozoa contains high amounts of polyunsaturated fatty acids making them vulnerable to lipid peroxidation and ROS-induced damage [11,12]. Oxidative stress may also affect the integrity of DNA, resulting in high levels of DNA fragmentation [13]. Furthermore, oxidative stress is associated with increased apoptosis [[14], [15], [16]]. These pathological pathways are correlated with a range of negative clinical outcomes like damaged germ cells, impaired fertilization, increased miscarriages and enhanced risk of disease in the next generation [17,18]. Although the literature agrees on the definition of oxidative stress, no consensus exists concerning its classification, hereby differentiating between physiological and pathological levels.

Knowledge of ROS and their potential pathology are taken into consideration when studying processes in the field of assisted reproductive techniques (ART). Here, the most viable sperm are selected for use in in vitro fertilization (IVF), intracytoplasmic sperm injection (ICSI) and intrauterine insemination (IUI) [19,20]. IUI, often used in male infertility, is carried out by injecting small volumes of prepared semen directly into the uterine cavity. IVF is performed by putting together retrieved oocytes and spermatozoa on a culture dish to achieve fertilization [20]. In case lower counts of motile sperm can only be harvested, ICSI is used. This is when a single sperm is injected into an oocyte [19]. Outcomes can be measured in fertilization, clinical pregnancy, live births and miscarriages [21,22].

Current selection routines have been scrutinized due to the possible influence on ROS levels. Although the literature is contradictory, some methods might cause damage during the process [8,19,20]. In the meantime, new selection methods using alternative characteristics of spermatozoa, for instance their electrical charge or maturity, aim to target different quality parameters and thus to retrieve more suitable spermatozoa [23]. Moreover, they might be able to circumvent the possible damage that is done using some current selection routines, or, add to the quality of the selection when combining them [12,[24], [25], [26]]. Eventually both strive towards qualitatively better sperm for more successful fertilization rates and ongoing pregnancies [27].

Quality measurements using this DNA-fragmentation or lipid peroxidation might be able to give insights about the health of the sperm [28].

Multiple literature studies both in the area of physiology and assisted reproduction show an increasing depth of knowledge, even formulating novice ideas. Nonetheless, clinical implementation is restricted due to low quality evidence [21]. In order to clinically implement new knowledge of radicals and oxidative stress, formulating standardized measures of both oxidative stress and semen quality is crucial.

In terms of prevention, lifestyle is important in modern medicine. Well-known contributing lifestyle factors to oxidative stress include smoking, alcohol consumption, physical activity, psychological stress, drugs and diet.

This article aims to give an overview of the current literature, highlighting the potential of the relationship between oxidative stress and male infertility. Firstly, a brief overview of the physiology and pathology of ROS and oxidative stress is given. Then, the link with clinical medicine is highlighted in the shape of ART. Furthermore, we will discuss selection methods and the methods for detecting ROS. Lastly, we stress the importance of prevention by discussion contributing lifestyle factors. Eventually, this article emphasizes the relevance for clinicians to better understand and be aware of the relationship between oxidative stress and male infertility, putting scientific knowledge to use and improve clinical practice.

## 2 Physiology of reactive oxygen species

Reactive oxygen species are short lived reactive chemical intermediates with one or more unbound electron [6]. We speak of oxidative stress when there is an imbalance between the production of ROS and the antioxidant capacity [28]. Raised levels, exceeding antioxidant capacity, can be seen in living organisms under the influence of exogenous and endogenous sources [29]. Exogenous sources being radiation, inflammation, activation of oxidases and oxygenases, and loss of antioxidant capacity [6]. Endogenous sources include oxidative phosphorylation, peroxisomes and inflammatory cell activation [29].

At the correct balance, ROS play an important role in the bioregulation of cells. A few examples, not specifically related to male infertility, are their regulation of gene expression, cell adhesion and antibody production of leukocytes, and their involvement in the mechanism of ‘oxygen sensors’, e.g. in the carotid body, which are chemoreceptor cells that detect changes in the content of arterial blood [30]. Before elaborating on their relevance to sperm, a brief overview of their physiology follows.

### 2.1 Most relevant ROS

**Fig. 1:**
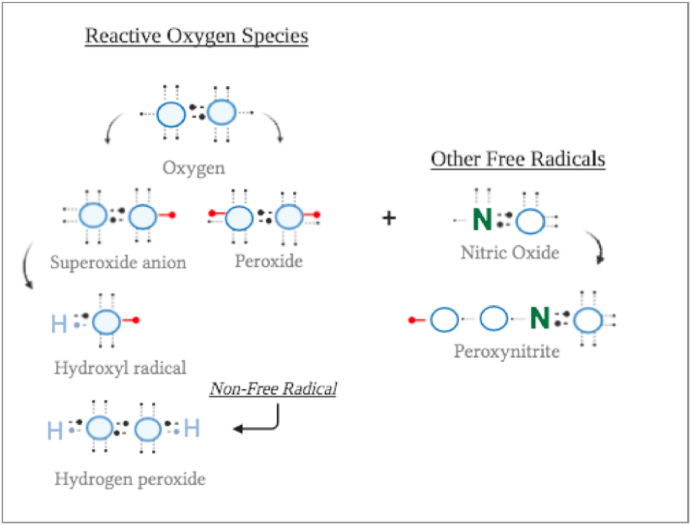
The most relevant ROS (=reactive molecules containing oxygen) or free radicals (=containing a free electron).

The term reactive oxygen species and free radicals are often used interchangeably. As one might now expect, this is not always the case. The umbrella term ROS is made up of free and non-free radicals. The distinction here being that free radicals specifically refer to molecules that contain unpaired electrons, making them extremely reactive. Both are shown in Fig. 1, which highlights the biologically important ROS. The largest part of ROS is due to the reduction of O_2_ to superoxide and peroxide. They become highly reactive as that reduction stops the electrons' spins from counterbalancing each other [30]. Metabolized superoxide products (e.g. hydroxyl radical (HO^•−2^) potentially cause even more damage as they become more reactive [6]. This type of ROS is known to be hydrophobic and has a low membrane permeability, so when produced within a cell's membrane can be detrimental to its survival [6]. The enzyme superoxide dismutase (SOD) catalyzes the above-mentioned reaction in most biological systems. Interestingly and relevant to this paper, seminal plasma is one of the largest sources of SOD [7].

Another category of oxidant molecules, that do fall within the definition of ‘free radicals’, are the biologically active nitric oxide (• NO), or ROS that react with nitric oxide to form peroxinitrite (ONOO^−^). Their unpaired electrons play an important role in the pathology of ROS as they damage lipids, proteins and nucleic acids [30].

Although much of this literature overview will cover the negative effects of ROS and oxidative stress, they definitely have some physiological features that need to be discussed. As their general function has been mentioned already, the next part will focus on the physiological relationship between ROS and spermatozoa.

### 2.2 Capacitation and the acrosome reaction

**Fig. 2:**
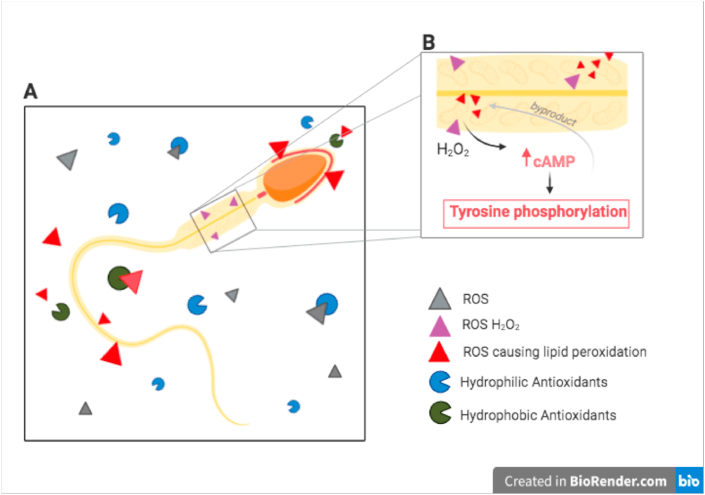
ROS and antioxidants surrounding a spermatozoon. b. Influence of H_2_O_2_ on mitochondria in the midpiece of the spermatozoa.

In the process of fertilizing an oocyte, spermatozoa undergo multiple changes called capacitation, after which the acrosome reaction follows. Both processes are influenced by tyrosine kinase [7,31]. The former process is initiated in the uterus or uterine tubes by substances from the female genital tract. During this complex process, glycoproteins and other seminal proteins are removed from the sperm's overlying plasma membrane and other changes within the membrane occur [32]. Then, the acrosome reaction commences as the capacitated sperm binds to a glycoprotein of the zona pellucida. The reaction triggers molecular changes that cause the perforations of the plasma membrane as well as the acrosomal membrane. This exocytotic process is linked to enzymes that facilitate fertilization like hyaluronidase and acrosin, a main feature is the acquisition of fusogenic ability [31,33]. Although Aitken (2016) clearly states there are still many gaps in the molecular pathway of capacitation, ROS seem to be a possible contributor [7]. Free radicals taking part in the capacitation of spermatozoa are probably generated by two sources: nicotinamide adenine dinucleotide phosphate (NADPH) oxidase located in the plasma membrane, and the mitochondrial nicotinamide adenine dinucleotide (NADH)-dependent oxido-reductase, however the contribution of the latter is still unclear. It has been shown that next to O_2_^−^ and H_2_O_2_, also nitrogen reactive species such as a ^•^NO and ONOO− are crucial for the process. The O_2_^−^ has been proved to react with ^•^NO and lead to the formation of ONOO−, which is responsible for the oxidation of cholesterol to oxysterols. The oxysterols efflux from the plasma membrane enhances its fluidity which is important for the acrosome reaction and the fussion of spermatozoa with the oocyte. Amongst other mechanisms, ROS (combination of the ONOO− and H_2_O_2_) has shown to be involved in the inhibition of tyrosine phosphatase activity [34]. As shown in Fig. 2b, H_2_O_2_ causes large production of cAMP, which leads to protein kinase A-dependant tyrosine phosphorylation of target proteins involved in sperm-oocyte interaction [29]. When adding H_2_O_2_ to human sperm, the enhancement of tyrosine phosphorylation leads to over-capacitation and a state of oxidative stress [7]. This oxidative phosphorylation occurs in the inner mitochondrial membrane and generates excessive amount of ROS as byproducts [29]. Firstly, these byproducts may cause lipid oxidation decreasing sperm motility and their ability to fuse with the vitelline membrane of the oocyte. Secondly, they damage sperm DNA and RNA [29] and cause the sperm to initiate apoptosis. Mitochondria are both a source of ROS and very susceptible to their potentially negative effects. Mitochondria entail less DNA-repair mechanisms than nuclear DNA and thus their mutation rate is estimated two times higher. These mutations then interfere with the cells apoptotic mechanisms, leading to more apoptotic spermatozoa in infertile males, compared to fertile males [29].

### 2.3 Antioxidants

The effects of ROS on certain pathways in sperm capacitation can be reversed or prevented by antioxidants [7]. As a self-defense and protective mechanism, human seminal plasma contains antioxidants that roam for ROS [8]. It is noteworthy that spermatozoa themselves do not have much antioxidant enzymes, and that their protection leans on the antioxidant systems present in the semen [35].

The seminal antioxidants are either enzymatic or non-enzymatic. The enzymatic group contains superoxide dismutase (SOD), catalase and peroxidase that remove ROS through catalytic activity [35]. The non-enzymatic groups, which are low molecular weight compounds, scavenge and inactivate any type of ROS produced by metabolic activity [36,37]. The most common examples are glutathione, uric acid and coenzyme Q10 [38]. The non-enzymatic group can be further split into hydrophilic and hydrophobic groups. The former being primarily present in blood serum, extracellular fluid and seminal plasma. Whereas the latter protects membranes from ROS-mediated lipid peroxidase, as illustrated in Fig. 2a [38].

Earlier research describes the spermatozoa's difficulty protecting its tail and membrane from lipid-peroxidation [35]. In accordance with this, a recent study on water- and fat-soluble antioxidants showed hydrophilic antioxidants to be the main defense mechanism [38]. This suggests more research should be conducted on the role of hydrophobic antioxidants, and whether their supplementation might be beneficial to help prevent damage from excessive ROS.

Deficiencies in both enzymatic and non-enzymatic antioxidants have shown to lead to oxidative stress. A combination of adding multiple antioxidant supplements in vitro, have shown to be effective improving sperm parameters. This is due to the antioxidants being able to target the exogenous ROS from, for instance, leukocytes. Unfortunately, this does not apply to the DNA damage done by ROS [29]. The endogenous ROS, produced mostly from the mitochondria, cannot be targeted by in vitro supplemented antioxidants [39]. Here, systemic antioxidant therapy and lifestyle alterations might be able to make the difference, as they could function as an earlier intervention for preventing DNA damage.

## 3 Pathophysiology

### 3.1 Influences of ROS on the cellular level

#### 3.1.1 Lipid peroxidation

While different animal studies determined the susceptibility of lipid peroxidation in animal spermatozoa, Jones et al. were the first to highlight the susceptibility of oxidative stress induced lipid peroxidation in human spermatozoa [11]. Human sperm plasma membrane phospholipids have an abundance in polyunsaturated fatty acids (PUFA). PUFAs contain various double bonds separated by methylene groups, which are sensitive in generating lipid peroxides and aldehydes, associated with reduced motility, viability, structural integrity, and metabolic activity of human sperm [11,12].

**Fig. 3:**
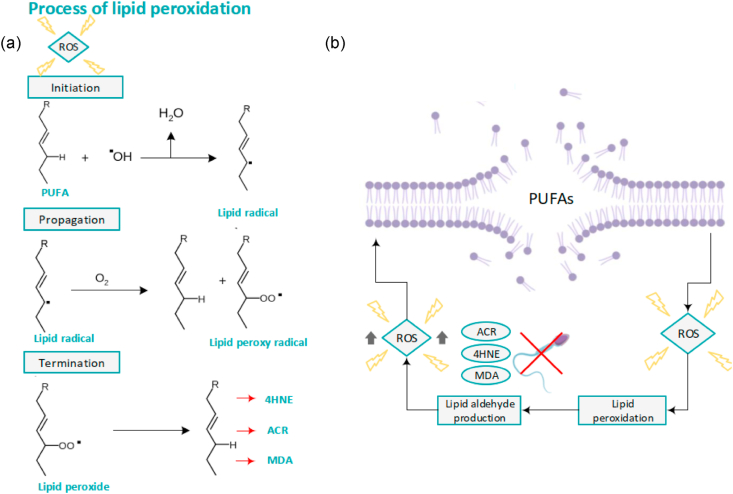
Lipid peroxidation (a) The lipid peroxidation cascade explained in three stages, called initiation, propagation, and termination. (4-hydroxynonenal (4HNE), malondialdehyde (MDA), and acrolein (ACR)) (b) Lipid peroxidation causes a generation of various electrophilic lipid aldehydes like ACR, 4HNE and MDA. Those electrophiles reduce the function of spermatozoa and do cause even more generation of ROS.

Lipid peroxidation means the oxidative degradation of lipids. Free radicals ‘steal’ electrons from the lipids in sperm cell membranes, creating cell damage such as changes in structure, accumulation and dynamics of lipid membranes; leading to a free radical chain reaction mechanism, causing highly reactive compounds [40]. The lipid peroxidation cascade can be explained with three stages, called initiation, propagation, and termination of which each process is depicted in Fig. 3.

During the initiation phase, high levels of ROS activate the liberation of PUFAs [40]. For example, hydrogen atoms from the PUFAs of human sperm plasma membranes will be isolated. Once combined with hydroxyl radicals (OH*-), water is generated, along with lipid radicals [12]. The propagation stage is characterized by the reaction of unstable lipid radicals with oxygen (O_2_). This generates peroxyl radicals [17]. To stabilize, these molecules have a tendency to further react with hydrogen atoms from the lipids in the sperm plasma membrane. This chain reaction mechanism leads to a continued damaging lipid peroxidation [41]. A stable end product is formed when the termination phase arises. Due to reactions of two radicals, non-radical species are created. This occurs only when the concentration of radicals is high enough to cause radicals to collide. Various electrophilic lipid aldehydes like 4-hydroxynonenal (4HNE), malondialdehyde (MDA), and acrolein (ACR) are created which will terminate the reaction [11,42]. 4HNE, MDA, and ACR are powerful electrophiles, forming adducts with several proteins in sperm cells and generating reduction in spermatozoa's function (See Fig. 3 (b)). Binding to proteins in the mitochondrial electron transport, leads to an electron efflux. These electrons react with O_2_. This causes a generation of even more (mitochondrial) ROS [43] (See Fig. 3 (b)). Another study showed the impact, specific on 4HNE. They conclude that 4HNE affects sperm function via epigenetic modifications and reduced sperm motility [44].

#### 3.1.2 DNA damage

Oxidative stress may also affect the integrity of DNA, resulting in abnormal sperm function. Male infertility has been associated with the presence of high levels of DNA fragmentation in spermatozoa compared to fertile men [13]. Excessive levels of ROS linked with a decreasing level in antioxidants can lead to oxidative stress, and subsequently generate nuclear and mitochondrial DNA damage [3,13]. Mahfouz and his colleagues studied DNA fragmentation between a group with normal seminal ROS levels (<250 relative light units/sec/10^6^ sperms) and high seminal ROS levels (>250 relative units/sec/10^6^ sperm). Findings stated that an increase of 25% in seminal ROS levels leads to a 10% increase in DNA fragmentation [45].

DNA damage is represented in different forms of modifications such as DNA single or double-strand fragmentation, the creation of abasic sites, changes in purine, pyrimidine, deoxyribose, and DNA crosslinking [46]. These modifications can result in starting or stopping gene transcription, increased degradation of telomeric DNA, epigenetic alterations, replication errors and GC to TA transversions [3,46]. ROS creates oxidized DNA base adducts within the DNA strands, like 8-hydroxy-2′-deoxyguanosine (8-OHdG), which affects the integrity of sperm chromatin [47]. Infertile men often exhibit poor chromatin packing compared to fertile men, making sperm DNA vulnerable to oxidative stress [48].

Spermatozoa do only have one base excision repair (BER) enzyme upstream in the frame reading process, responsible for DNA damage repair. This enzyme is called 8-oxoguanine DNA glycosylase 1 (OGG1), which is responsible for the release of adducts into the extracellular space, due to the excision of DNA base adducts [46,49]. Spermatozoa do not have BER enzymes like apurinic endonuclease 1 (APE1) and X-ray repair cross-complementing protein 1 (XRCC1). For this reason, the DNA repair capacity of spermatozoa is fragile, which leads to a compromised repair of oxidized DNA base adducts, like 8-OHdG [49]. Furthermore, it has been reported that 8-OHdG induces germline mutations, indirectly leading to DNA fragmentation in human spermatozoa [50].

#### 3.1.3 Apoptosis

**Fig. 4:**
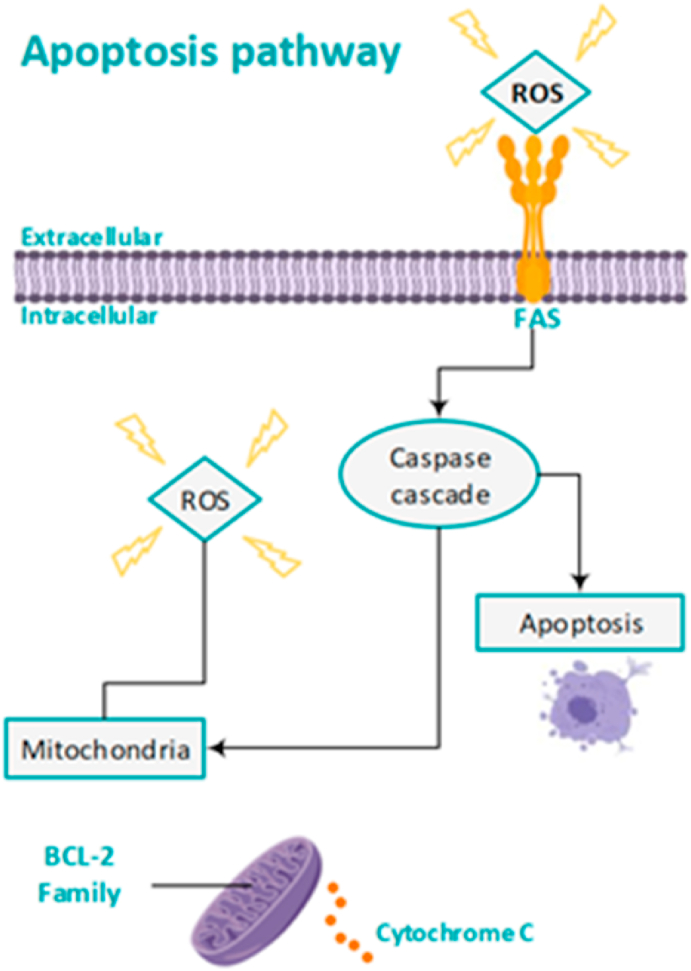
Mechanism of ROS-mediated activation of the apoptosis pathway.

Apoptosis is a physiological phenomenon characterized by cellular biochemical and morphological modifications that takes care of controlled cell death [41]. High amounts of oxidative stress activate the apoptosis pathway [51] (See Fig. 4). During the early development of testicular precursor germ cells in the seminiferous epithelium of the testis, apoptosis plays an important role in regulating the germ cell to Sertoli cell ratio, which is an indication of the number of germ cells supported by each Sertoli cell [41,52]. Moreover, it plays a vital role in the spermatogenic stages in the testis, ensuring that defective germ cells go into apoptosis and do not differentiate into mature spermatozoa [52]. Those ultimately define the extent of male fertility. The apoptotic pathway consists of highly intricate, sophisticated, and energy-dependent cascade mechanisms. These are regulated by two key modulators, called the intrinsic mitochondrial pathway and the extrinsic Fas receptor complex pathway.

Activation of the intrinsic mitochondrial pathway leads to the release of cytochrome-C from the inner and outer mitochondrial membrane. Cytochrome-C further activates the apoptotic caspase cascades which causes apoptosis. An important regulator in this pathway is the Bcl-2 family, which can both block and promote the apoptosis caspase pathway [41,53]. An example is the protein BAD, which is a Bcl-2-associated death promoter. Spermatozoa are prevented from entering the intrinsic apoptotic pathway due to the activity of the enzyme phosphatidylinositol 3-kinase (PI3K). PI3K activity ultimately leads to phosphorylation of the BAD protein, whereby the spermatozoa prevail in the apoptotic phase and survive [14]. Higher amounts of oxidative stress lead to dephosphorylation of BAD and causes a pro-apoptotic state. This change is also known to create pro-apoptotic pores in the outer mitochondrial membrane, which further activate the intrinsic apoptotic cascade. The extrinsic, also called the cytoplasmic pathway, is triggered through the Fas death receptor. Fas is a type of receptor protein which is a member of the tumor necrosis factor (TNF) family [54]. When a death stimulus activates the pathway, the Fas ligand at the membrane activates the Fas complexes which triggers the apoptosis pathway [52].

The apoptotic cascade can be activated as a result of excessive oxidative stress and ROS production [55]. A study of Wang et al. showed higher levels of cytochrome C, and higher levels of activated caspase 3 and caspase 9 enzymes in infertile men, which are all proteins mediating apoptosis [15]. Increased levels of ROS can cause oxidation of the mitochondrial pores and causes releasing cytochrome C due to disruption of the mitochondrial membrane [51]. Furthermore, high levels of ROS triggers Fas death receptor causing apoptosis via the extrinsic pathway [56]. The induction to an apoptotic cascade is characterized by rapid loss of spermatozoa motility, even more generation of ROS, caspase activation in the cytosol, and oxidative sperm DNA damage [14,15]. The former is in line with a study, showing that the apoptotic process, triggered by oxidative stress in the male genital tract is the main pathway leading to DNA damage [16].

### 3.2 Influences of ROS at the clinical level

When an imbalance between ROS generation and antioxidants arises, it could importantly play a role in male subfertility. Several factors, such as lipid peroxidation, DNA damage and apoptosis have been identified to link ROS production with male infertility, however, the direct link is still missing as a result of limited evidence due to significant heterogeneity in current research results. What is known is the results of studies in in vitro settings and not the natural environment, which bears the risk of measuring ROS because of the technique itself [17]. Although increased miscarriage rate is seen in patients with high DNA damage compared with those with low DNA damage as described in cohort studies, the true challenge is to choose the right spermatozoa for the fertility treatment [15,17,57].

#### 3.2.1 Assisted reproductive techniques

As a general principle for ART, the most viable sperm should be chosen for insemination. Here, it could be important to understand the oxidative state of an infertile male's sperm. If high levels of ROS prevail, we might be able to speak of chronic oxidative stress. It could be worth implementing treatments (eg. monitored lifestyle changes) and seeing what this does to the ROS levels. Yet, without standardized cut-off scores, conclusive statements remain unattainable.

When it comes to our knowledge of oxidative stress in the process of ART, two important factors prevail [58,59]. Firstly, neat sperm, from the ejaculate, is processed for use in the laboratories. Ironically, some sperm processing in the lab might interfere with ROS. Some articles claim that the processing of sperm, before it is used for artificial insemination, like washing and centrifuging, increases ROS [58,[60], [61], [62]]. However, other authors found that processing semen is beneficial for its oxidative state. This is mainly due to the elimination of ROS producing leukocytes [58,63]. The discrepancy in results may be due to the fact that no consensus exists for a standardized way to measure ROS or oxidative stress. Moreover, whilst clinically the silica-based density gradient seems to be used most as standard routine, laboratories have also been testing alternative methods to select sperm [64,65].

Secondly, it is necessary to define what makes sperm viable. What measurements or parameters are used to assess the quality of sperm, and how can these be linked to ROS or oxidative stress? By linking ROS to various parameters, scientist may be able to comprehend their effect on outcomes of ART.

This section will attempt to elaborate on both topics. It starts off by giving a brief overview of contemporary methods for sperm selection with respect to ROS. Then, it will focus on parameters used to determine the quality of sperm.

#### 3.2.2 Sperm selection methods

For evaluation of ‘natural’ or normal ROS levels in semen, it is best to use unprocessed semen as it is after ejaculation [62]. According to Moein et al., although this sperm will not be used for ART, it gives the best clinical indication of the oxidative state in semen. *In vivo*, sperm selection in the female genital tract obtains potential healthy motile sperm and eliminates material such as seminal plasma [58]. For the use of ART, this process is mimicked as much as possible by selecting healthy spermatozoa in vitro. Here spermatozoa are also separated from materials that would normally left behind in the vagina. These materials are immature spermatozoa and spermatozoa with low to absent motility, and seminal plasma proteins, bacteria and cell debris (eg. epithelial cells and leukocytes) [65].

##### 3.2.2.1 Density gradient techniques

A very routinely used method for sperm processing, in fertility laboratories, is density gradient centrifugation (DGC) [65]. Here, semen is placed on top of a higher density medium. In principle the separation is based on (nuclear) density, but usually after centrifugation the soft pellet at the bottom is enriched in highly motile sperm since many non-vital and damaged spermatozoa will stuck at the gradient's interface. Compared to the swim-up method (mentioned below), this method is better at selecting spermatozoa from semen with a low sperm count [58]. Despite the successful usage in clinical practise Percoll ®, a broadly used gradient material was taken off the market for use in ART. The reason was concerns over membrane alterations, inflammatory responses, and adhering to the sperm membranes. However, to date there is no convincing evidence to support these concerns. As Percoll® was removed from the market, the alike silica medium stabilized with covalently bound hydrophilic silane, was introduced, and is now used to date under various names [65]. Though not many studies have compared various media, one article says that the potential difference in DNA fragmentation can be related back to the donor rather than the used medium.

Another aspect of washing sperm is that it eliminates those seminal factors, mentioned above, that contribute mostly to the production of ROS. For instance, by eliminating these materials in neat sperm with high ROS levels, DGC is able to eliminate up to 81.56% of ROS [63]. It is also noteworthy that centrifuging sperm also eliminates the sperms protective agents against ROS [63].

Making further improvements on DGC is strenuous as a lack of evidence with equal measures, methods and outcomes prevails [65].

##### 3.2.2.2 Swim-up

Another conventional and frequently used sperm selection method in fertility laboratories is the swim-up method [27]. This process highlights the sperms motile characteristic [65]. It involves spermatozoa to move actively from a pre-washed pellet into an overlying medium. It has great fertilization success through ART for female subfertility, however, fails to reach the same success in male subfertility due to less motile sperm from the ejaculate in combination with the low yield of the procedure [58]. A grave limitation to this method is that, by sedimenting, motile sperm can come in close contact with high ROS producing leukocytes and cell debris [58,66]. This puts initially healthy spermatozoa at high risk of damaging their membranes through lipid peroxidation [58].

##### 3.2.2.3 Alternative sperm selection methods

The above-mentioned methods are used routinely in clinical practice. In order to keep improving the quality of yielded sperm for ART, scientists have started to look at alternative characteristics. They hypothesize that these characteristics may eventually be able to improve sperm selection, thus improving ART outcomes. The following section discusses electrophoresis, the non-apoptotic sperm selection and the membrane maturity method (based on HA-binding).

##### 3.2.2.4 Electrophoresis

The electrophoresis-based method (the electrical charge of spermatozoa) has been proposed to filtrate viable sperm cells with normal morphology and intact DNA. The perks of this method are that it avoids additional production of ROS as it is able to eliminate leukocytes and immature spermatozoa which act as major source of ROS [58,59]. Unfortunately, this method fails to increase or improve the cells motility, as DGC does [67]. Besides this, it is very costly which might prevent it from being implemented in andrology laboratories [59].

A cheaper alternative is the zeta potential method. This allows for the negatively charged sperm to adhere to the positively charged tube, after which it is centrifuged to dispose of the non-adhering cells. The zeta potential method, compared to the DGC, was found to select sperm cells with higher DNA integrity. Regrettably, its use is limited in oligozoospermic samples due to low sperm recovery rates [59]. The results of using just electrophoresis are very limited in sample size and differ between studies. They fail to find a significant difference between DGC and electrophoresis. However, when comparing DGC to the zeta method combined with DGC, fertilization rates were significantly higher in the latter group [59]. Here, it might be interesting to take ROS or DNA-fragmentation as parameter. If the combined methods manage to minimize ROS damage, circumventing additional DNA fragmentation, they might be able to significantly improve pregnancy outcomes. Only with these sorts of considerable results, can the clinical implementation of this method be deemed.

##### 3.2.2.5 Non-apoptotic sperm selection

The non-apoptotic sperm selection is based on membranous phosphatidylserine being an indicator of early apoptosis. This binds with Annexin-V conjugated paramagnetic microbeads. It is then run through a magnetic-activated cell sorting system which lets non-apoptotic cells flow through freely [68,69]. In order to get rid of unnecessary and harmful byproducts DGC is used [70]. The combination of the non-apoptotic sperm selection with DGC resulted in 30% less DNA damage in spermatozoa, compared to solely using DGC [71]. As DGC alone filters out 50% of damaged sperm, it seems a very valuable tool for ART [59,72]. Although the promising percentages from above are from healthy donors and men with unexplained infertility, the direct link with ROS was not made. Indirectly, we can expect sperm with high ROS to show more DNA fragmentation. However, studies that link ROS, DNA fragmentation and this sperm selection method to one another are yet to be conducted. Nevertheless, these findings might be beneficial for patients presenting with high DNA-fragmented sperm. Although some positive results are described in case studies, sample sizes have remained insufficient and better controlled clinical trials are yet to be conducted [59,64].

##### 3.2.2.6 Membrane maturity

Including spermatozoa can also be done based on their maturity. Mature sperm present with hyaluronic acid binding sites and the testis-expressed chaperone protein HspA2, during cytoplasmic extrusion [73]. These mature sperm cells can be collected by adding hyaluronic acid to a petri dish, binding mature spermatozoa [59]. The likelihood of attaining >14% of motile and morphologically intact sperm (based on the Tygerberg strict criteria) increases 3-fold with this method [74]. Furthermore, research suggests that HA bound spermatozoa has significantly less DNA fragmentation than DGC selected ones [75]. From these results it might be possible to see HA binding as a potential selection method with minimal ROS damage during sperm handling.

However, from the results of a Cochrane review, this method is far from clinical implementation as results failed to show improvement of ART outcomes (live births and clinical pregnancy rates). Nevertheless, the review did find a slightly decreased rate of miscarriages after use of HA binding, (routine ICSI 20% miscarriage per clinical pregnancy, HA-ICSI ranging from 9% to 16%) though they remain critical of the quality this evidence has [64]. The latter could be in line with the idea that HA binding selects more mature sperm cells with less DNA fragmentation, possibly decreasing the risk of miscarriage. Further research should be conducted to look into this method in relation to specific donor groups (high or low DNA fragmentation) and ART outcomes. Preferably so that randomized controlled trials (RCT's) with high quality evidence can be described.

In sum, the evidence that these alternative methods significantly improve clinical outcomes is weak and controversial [64]. For this reason, it cannot be implemented as a clinical routine. However, these methods do consider alternative characteristics of spermatozoa which might eventually give clinicians more information about the sperm quality. It remains unclear whether these methods might be more beneficial for males with for instance higher DNA fragmentation rates [64]. Combining them with extensive research into their relationship with ROS and oxidative stress, the methods might be able to offer new insights in the correlation between spermatozoa, ROS, and their ART outcomes.

#### 3.2.3 Anti-oxidant interference

It is clear that ROS, or oxidative stress, probability have a negative influence on spermatozoa function leading to a negative outcome of ART. Besides considering antioxidant therapy for subfertile males, solutions must be sought to minimize potential damage during the handling of sperm for ART. In line with this, literature suggests adding antioxidants to the sperm preparation media [76,77]. Taherian et al. (2019) researched the effect of adding alpha-lipoic acid (ALA), a strong antioxidant, to the washing medium. ALA is said to create a robust shield and protects the membrane from ROS. Amongst other mechanisms, ALA can scavenge free ROS and modulate endogenous antioxidants. Adding ALA allows spermatozoa to maintain their motility and viability after centrifugation and upon culture [76]. Another study added ethylenediaminetetraacetic acid (EDTA) and catalase to the sperm preparation medium. Both showed lower levels of ROS, when measured by a chemiluminescence assay. Additionally, this study found a reduced DNA fragmentation rate (measured using the comet assay) after adding antioxidants to the medium. The application of EDTA also improved forward motility of spermatozoa. No beneficial link was found for improving lipid oxidation [77]. The inclination here is that an improved DNA fragmentation would improve clinical ART outcomes. The direct link between the use of in vitro antioxidants in the sperm selection routine and ART outcomes such as fertilization rates and successful pregnancies, is yet to be researched. Nonetheless, as also briefly mentioned in the physiology section, an in vitro antioxidant solution must not distract from the importance of preventing abundant ROS prior to infertility treatment. This can be done through lifestyle interventions such as the cessation of smoking, exercise, weight loss, and a varied diet containing natural antioxidants [78].

## 4 Lifestyle

**Fig. 5:**
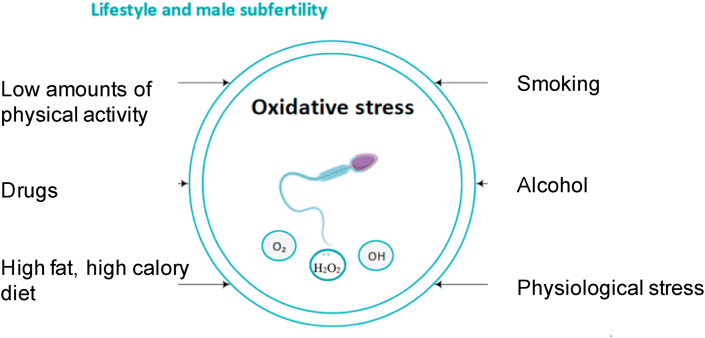
Lifestyle factors which negatively affect the fertility of males.

Different lifestyle factors are to the best of our knowledge known as high effectors in human health (See Fig. 5). Several studies have been performed on lifestyle and male subfertility. Various lifestyle factors are presumed to have a detrimental effect on male fertility due to an imbalance between the production of ROS and the protective effect of antioxidants, leading to oxidative stress. The detected oxidative stress in the semen of infertile men are more likely a result of increased ROS production, rather than lower antioxidant levels [9].

### 4.1 Smoking

Approximately one third of the world's population daily use tobacco products, with Europeans as the highest tobacco users [[79], [80], [81]]. Cigarette smoke contains high amounts of hazardous substances, including nicotine, cotinine, hydroxy cotinine, alkaloids and cadmium, lead, and other carcinogenic compounds. These substances affect the oxidative/antioxidant balance, whereby ROS is increased, and antioxidant levels are reduced [41,79,82].

A recent meta-analysis of twenty articles, using the WHO 2010 methods for sperm analysis, concluded there was a significant negative effect of smoke on sperm parameters like sperm count, motility and morphology. Those negative effects are most significantly seen in infertile men, although, no significant effect on semen volume has been seen. The negative effect of smoking cigarettes is dose dependent. One can differentiate the effect on semen quality particularly in moderate (10–20 cigarettes/day) and heavy smokers (>20 cigarettes/day) [79].

Cigarette smoking also leads to DNA damage, due to an overload in human spermatozoa ROS. It can cause abnormalities in histone-to-protamine transition, disturbs the expression of micro-ribonucleic acids (miRNAs), and can reduce protein phosphorylation. Those do all have a major impact in gene regulation, causing complicated fertilization [82,83]. Apart from the human studies we have discussed so far there are also important insights into this topic from animal experiments. In rats exposed to cigarette smoke, higher amounts of 8-OH-dG in blood have been measured, also indicating DNA damage [84]. Additionally, consuming cigarette smoke contributes to lipid peroxidation and raises the oxidative stress in the testis. A study from Oyeyipo et al., 2014, treated rats 30 days orally with nicotine and showed elevated testicular lipid peroxidation leading to an increase in oxidative stress. Also decreased testicular antioxidant levels have been detected [85]. Both indicate the reduction of spermatogenesis in rats due to increased ROS production. The midpiece of spermatozoa contains mitochondria, which generate chemical energy in the form of adenosine triphosphate (ATP). ATP plays an important role in sperm motility. Gogol and his colleagues showed a reduction in ATP levels due to increased lipid peroxidation [86]. Since ROS leads to lipid peroxidation, cigarette smoke can indirectly affect ATP levels. Additionally, creatine kinase (CK) is an energy reservoir for buffering and rebuilding adenosine triphosphate (ATP). In smoking males, the CK activity in spermatozoa is lower, which may potentially impair sperm energy homeostasis and especially affect sperm motility [87]. Therefore, smoking can indirectly be seen as a causative factor in male subfertility.

Smoking elicits a chronic inflammatory response, which has been associated with a 48% increase in seminal leukocyte concentrations, and a 107% increase in reactive oxygen level [80]. The smoking caused inflammatory response recruits proinflammatory leukocytes into semen plasma. Since leukocytes have shown to be one of the main sources of ROS, it relates to male subfertility [88]. Furthermore, a study showed decreased zinc concentrations in smoking people [83]. Zinc plays an important role as anti-inflammatory and antioxidant agent, causing a disparity in the redox balance.

Besides conventional smoking, new delivery devices for consuming tobacco and its derivatives arise and are being used more and more frequently, for example, e-cigarettes. Those do have other constituents than traditional cigarettes, although most still contain nicotine. A cross-sectional study of men from the general population found influences in semen plasma, using e-cigarettes. Significant reductions in sperm count and concentration have been measured [81]. This upcoming tobacco use, most seen as substitutes of the conventional cigarettes have often been seen as less harmful. Future research should be done to find causal relations in the upcoming other types of tobacco products.

### 4.2 Alcohol

Alcohol consumption is widespread in the western world [89]. Little evidence has been found about direct mechanisms of alcohol-induced oxidative stress, as a cause of male subfertility. Metabolization of ethanol produces acetaldehyde, subsequently creating free radicals in the human body. Changes between the production of ROS and the antioxidant system has been linked to the ethanol-metabolism [90]. Evidence in the literature shows that alcohol-consumption is associated with ROS production within the body, mainly in the liver. Besides, scientists also suggest that alcohol consumption causes ROS production, contributing to male subfertility [91].

Two animal studies concluded an effect of alcohol consumption and oxidation in semen. Alterations on the membrane and end products of lipid peroxidation, like MDA on testis have been found [92]. They are in line with research that shows increased lipid peroxidation levels as well as increased testicular antioxidant levels [91]. These factors provide evidence of ethanol-induced oxidative stress within the testis.

Alcohol consumption is also associated with changes in semen parameters. A recent meta-analysis of 16,395 men in 15 cross-sectional studies, determined that occasional alcohol consumption does not adversely affect semen quality. Daily alcohol consumption significantly showed decreased semen volume and sperm morphology. It has to be noted that the 15 included studies all measure the amount in alcohol consumption differently. Another study even determined a positive effect in semen quality after moderate alcohol (4–7 units/week) consumption [93]. When treated with alcohol, rats showed significant reduction of sperm motility and concentration of sperm in their testis [91]. The inconsistency between the studies could be due to different categorization and differences in intensity of alcohol consumption, or other lifestyle factors. This manifests the need of more scientific studies evaluating the effects of alcohol-induced changes in semen quality on reproduction.

### 4.3 Physical activity

It is well known that physical exercise contributes to human health, keeping the body fit and ensuring a well-functioning immune system [94]. Regular exercise enhances antioxidant protection in the human body, although exhaustive exercise could have deleterious effects and cause oxidative stress. The exact redox mechanisms still remain unclear, but the NAPDH oxidase, xanthine oxidase and mitochondria seem to be the potential contributors [95]. Still, more research needs to be done about physical activity and their affect in male fertility, since there is limited evidence with contradictory results.

Physical inactivity has been associated with increased oxidative stress in the body [96]. Decreased sperm concentration and sperm count was found to be associated with increased television viewing [97]. Watching television for more than 20 h a week showed 44% lower sperm concentration compared with men who rarely watched television. Those indicate a negative effect of a physically inactive lifestyle and their effect on male fertility, possibly due to ROS production and decreased concentrations of antioxidant scavengers.

Perez et al., 2019, compared 32 studies, suggesting a beneficial effect in doing exercises and semen parameters of men. They found a positive correlation between moderate and/or high physical activities of 20–80 METs-h/week^1^ and semen parameters. Those are in line with a recent randomized controlled trial (RCT) that showed positive results on semen parameters with resistance exercise training, which can be seen with physical activity of 40–80 METs-h/week. They further showed positive results on DNA integrity, anti-inflammatory factors as well as the antioxidant activities measured in semen plasma [98].

Nevertheless, excessive duration and heavy physical exercise could possibly have negative effects. A meta-analysis of 333 men, showing the effect of exhaustive physical exercises (>80 METs-h/week) suggested detrimental effects on semen parameters. It has the most significant detrimental effect on sperm motility. Besides, sperm morphology and volume were decreased as well [99]. Those impairments of semen parameters are likely to be related to peroxidative damage in spermatozoa due to increased oxidative stress [100]. A prospective study comparing low and moderate physical activities with exhaustive exercises in 108 humans showed significantly higher levels of 8-OH-dG, MDA, ROS and reduced antioxidant levels [101]. Intensive cycling training (16 weeks) in young healthy men affected the seminal oxidant/antioxidant ratio. Levels of ROS and MDA increased while reduced levels of antioxidant where found. Even low-to-intensive cycling training shows negative effects in spermatozoa [102]. Both RCTs found reduced sperm quality parameters, DNA damage, and elevated oxidative stress production in sperm [103].

One underlying mechanism could be the increasing temperature during exercise, generating oxidative stress. It has been concluded that male cyclists do have increasing testicular heat [103]. Cyclists do wear a lot of tightp-fitting lower body clothing, which can contribute to higher scrotal and testicular temperatures [104]. Rao et al., 2016, concluded scrotal hyperthermia effects; determining detrimental effects in human sperm DNA integrity, sperm protein expression, and sperm apoptosis, which are all indicators of ROS generation [105]. Both studies showed that elevated scrotal temperatures, possibly caused during exercise, could produce excessive generation of ROS which do have deleterious effects on male fertility. The use of anabolic androgenic steroids (AAS) could also be an underlying mechanism to the reduction in male fertility health. AAS use has been related to weakened sperm quality. Abstinence from ASS supplementation does significantly increase the median sperm concentration, that was 2.6 (95% CI: 1.1–5.8) times higher than the baseline median sperm concentration [106]. A recent study showed that the use of AAS is positively related with the number of hours participants exercised per week. Males who exercise more than three times a week in the gym are significantly associated with a higher use of anabolic steroids for performance improvement [107].

The exact mechanisms and contributing factors of physical activity is still a debated issue. Outcomes are different due to differences in the activity's intensity, type, and oxygen supply during sport. More research is required in this area, specifically about different sports and at which intensity they are likely to generate deleterious amounts of oxidative stress causing male subfertility and which exact underlying mechanisms are causing the reduction in male fertility health.

### 4.4 Psychological stress

Psychological stress, a feeling of emotional strain and pressure is prominent in any society. It is linked to increased levels of cortisol, epinephrine and norepinephrine (NE) and has also been related to male subfertility [108]. A study showed detrimental effects in semen parameters [109,110]. Two prospective studies have linked psychological stress to sperm quality in healthy medical students during an exam period. Their psychological stress was measured by a widely used State Trait Anxiety Inventory questionnaire and students with other stress factors, besides the exam period were excluded. Eskiocak et al., 2005, showed a reduced sperm quality and reduced antioxidant levels [111]. Sperm motility has been reduced compared to three non-stressful months after the examination period. Another study, similar in protocol, showed significantly lower sperm motility, sperm concentration and seminal plasma arginase activity, whereby higher amounts of nitric oxide, which is a highly reactive free radical, were measured. Both studies showed reduced sperm quality, mediated by an imbalance between ROS and antioxidant levels [111,112]. Furthermore, sperm concentration, motility and morphology in healthy men who have had more than two recent stressful life situations, deteriorated [109]. This outcome is in line with a cross-sectional study of 1215 Danish men with idiopathic subfertility, who self-reported their psychological stress [113].

Recent studies show positive associations with doing yoga and meditation and reduced levels of ROS and nuclear and mitochondrial DNA damage, which enhances the sperm count and motility [114,115]. Mind-body practice, yoga and meditation have been linked to reduced levels of psychological stress with diminishing cortisol levels, anti-inflammatory cytokines, cell cycle control and modulating the immune response [41,114,115]. Furthermore, an association between sleep disturbances and sperm count, morphology and concentration are found by a Danish cross-sectional study of 953 healthy men [116]. A recent study showed positive correlation with the amount of sperm and progressive motility of sperm and the duration of sleep (0.249; 0.233; p < .05). They find that post-bedtime exposure to light-emitting digital media like the smartphone and tablet screens, are linked to reduced sperm quality (66). A new study investigated male reproductive health in people who are working discrepant against their inner biological clock, also called ‘circadian desynchrony’. They found that rotating shift work is related to lower sperm counts. They also found negative effects in sperm count non-work-related causes, like long time use of the mobile phone [117].

**Fig. 6:**
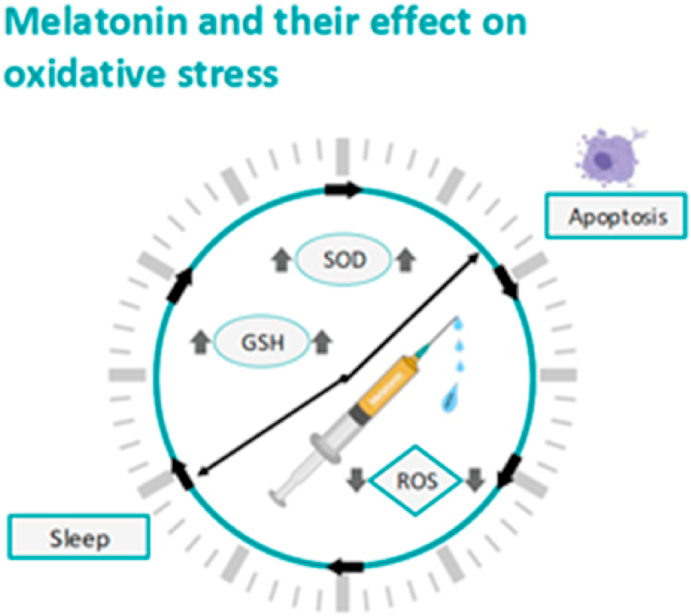
Melatonin injection plays a role in the sleep cycle and is seen as an antioxidant, exerting a protective role against oxidative stress and testicular cell apoptosis. Melatonin decreased ROS levels and increased the levels of GSH and SOD activities.

Melatonin injection, a hormone which plays a role in the sleep cycle, is seen as an antioxidant, exerting a protective role against oxidative stress and testicular cell apoptosis. Guo et al., 2017, showed positive results on sperm density, testicular oxidative stress and testicular cell apoptosis after injecting melatonin exposing them to restraint stress for five weeks in mice. The authors concluded that melatonin supplements strengthened the stress tolerance. Melatonin decreased ROS levels, increased antioxidants like glutathion (GSH) and superoxide dismutase (SOD) activities, and downregulated nitric oxide synthase (iNOS) and tumor necrosis factor- α (TNF-α) in stressed mice [110] (See Fig. 6).

### 4.5 Diet

Evidence shows the importance of nutrition and their effect on male fertility and influences in the amount of oxidative stress [[118], [119], [120]]. Drastic changes in society's nutrition pattern have taken place over the past decades. Most developing or developed countries eat the so-called ‘Western diet’. This diet consists particularly of hypercaloric, refined and nutritionally poor foods, characterized with high energy sugars, hydrolysed and trans fatty acids, omega-6 polyunsaturated fatty acids and processed foods, and further, low amounts of omega-3 polyunsaturated fatty acids, vegetables and fruits intake and vital micronutrients. This kind of unhealthy hypercaloric diet is associated with higher amounts of oxidative stress [118] A study showed a significant dose-response association between the dietary intake of saturated fat and reduction in sperm concentration and sperm count [119]. The highest quartile of saturated fat intake had 38% (95% CI: 0.1%, 61%) lower sperm concentration and a 41% (95% CI: 4%, 64%) lower total sperm count compared to men in the lowest quartile of saturated fat intake. Furthermore, reduced sperm concentration, motility, and an increase in DNA damage has been found in overweight and obese sub fertile men, which explains a link between BMI and oxidative stress [120].

A healthy diet could therefore be very important to keep the balance between the levels of ROS and antioxidant defense levels. Oostingh et al., 2017, showed a positive correlation between a strong adherence to healthy dietary pattern and semen quality of men. Higher sperm concentration, sperm count and progressive better motility were observed in a healthy diet pattern group, particular in men with a total motile sperm count <10 million spermatozoa, explaining a larger effect in sub fertile men (>10 million spermatozoa are seen as normal). Healthy diet pattern is perceived as eating less dairy, sugars, and saturated fat, while eating more healthy unsaturated fats, fruits and vegetables [121]. A cross-sectional study among 161 subfertile men, showed that dietary intake of fruits and vegetables are inversely associated with DNA damage of spermatozoa [122].

It seems that antioxidant supplementation improves DNA damage and is also associated with a better semen quality [118]. Antioxidants are very important as a self-defense and protective mechanism of oxidative stress [6]. Spermatozoa do not contain much of antioxidants, whereby most antioxidants are presented in the semen [35]. Most frequently used antioxidant supplementations, both alone and combined, include zinc, vitamin C and E, coenzyme Q10, carnitine, folic acid, and N-acetylcysteine [118,121]. Nenkova et al., 2017, showed lower amounts of zinc and selenium in the semen of infertile men compared to semen of men with normal fertility [123].

Further research on the relationship between nutritional factors and their effect in male subfertility is required. Their impact in oxidative stress production and therefore also reduced sperm quality and DNA damage are crucial to know. Improved knowledge about the exact role and efficacy of antioxidants, and better knowing which kind of diet and nutrition's will contribute the most to healthy sperm will improve male fertility.

## 5 Aging and oxidative stress in the male germ line

The aging process is defined as the progressive deterioration of bodily functions over time. One of the proposed hallmarks of this process is mitochondrial dysfunction, which is associated with an imbalance of the normal redox state of the cells, *i.e.,* the equilibrium between production of oxidant agents such as ROS and capacity of antioxidant systems [[124], [125], [126]]. In the male germ line, this imbalance is seen as a transition from the necessary regulatory roles of ROS during spermatogenesis [125] to the detrimental effects caused by an excess of these oxidant agents on fertility [127,128]. Accordingly, aging affects semen parameters and is related to high levels of DNA fragmentation in sperms [129,130].

The age threshold for the onset of defects in semen parameters was established by Stone et al. in 34 years old. By analyzing samples from more than 5000 men, these authors found that total sperm counts, and motility decrease immediately after 34 years of age, while other parameters such as sperm morphology are significantly affected beyond 40 years old [129]. In line with these results, a recent study showed that men older than 40 years of age have increased DFI (sperm DNA fragmentation index) in relation to men below this age [130]. The discussed causes for the obtained results include age-related excessive generation of ROS, sperm-limited antioxidant defences, and the stimulation of sperm damage by oxidative stress. Other studies have also obtained similar results from differently sized cohorts and age groups [131].

A retrospective study conducted by Pino et al. on more than 2000 men showed that parameters such as semen volume, sperm concentration, DNA fragmentation, and motility were differentially affected in three different age groups, *i.e.,* 31+, 41+, and 50+, with the oldest men showing the most negative results [132]. Not surprisingly, a similar study conducted by Paoli et al. showed that men between 51 and 81 years old have deficient sperm and semen parameters as compared to men between 20 and 32 years old, which is possibly related to an excess of ROS [133]. These authors also found that obesity, a known oxidative stress condition, contributes to alterations in sperm parameters such as motility and morphology and they did not find any association of smoking with semen quality. Additionally, at the molecular level, they observed a decrease in the levels of transcripts codifying for protamines, which could be related to alterations in chromatin packaging responsible for an increased DNA fragmentation due to oxidative stress sensibility. Indeed, the use of murine models has shown that aging affects chromatin integrity, making the sperm genome more prone to oxidative damage [134].

Murine models have been of great help in unraveling the effects of age-related oxidative stress during spermatogenesis since precursor germ cells cannot be easily obtained from patients. In this sense, a study conducted on four species of rodents, including rats and mice, reported that spermatozoa extracted from different regions of the epididymis, *i.e.,* with different maturation state, differ in their capacity of generating ROS both spontaneously and upon exogenous induction [135]. Precursor germ cells such as pachytene spermatocytes, round spermatids, and elongating spermatids also showed differential induced ROS production depending on the developmental stage. Additionally, spermatozoa spontaneously produced more ROS than the analyzed precursor cells. This can be related with the fact that spermatozoa have decreased antioxidant enzymatic capacity and increased ROS production during aging in rats [136]. In this murine model Weir and Robaire documented an age-dependent decrease in the activity of glutathione peroxidase (Gpx1, Gpx4) and superoxide dismutase (SOD) enzymes as well as increased levels of hydrogen peroxide and superoxide in epididymal spermatozoa. In line with these results, Robaire's group also showed that precursor germ cells display age-dependent susceptibility when responding to oxidative challenges, showing lower levels of transcripts for SOD, catalase, and peroxiredoxins than cells from young animals, which was associated with increased DNA fragmentation and elevated levels of ROS [137]. Finally, age-related susceptibility to oxidative stress is not restricted to spermatozoa and precursor germ cells. Leydig cells, the nurse cells that induce the entrance of precursor cells to the meiotic phase of spermatogenesis, show deficient antioxidant systems with aging, which may account for their age-dependent decrease in testosterone production [138].

Aging and its associated oxidative stress condition are main contributors to subfertility, infertility, and several disorders in the offspring of old males [139]. Even when a large number of studies can be found elsewhere highlighting associations between age, oxidative stress, and anomalies in the male germ line, there is a lack of mechanistic approaches addressing how the above-mentioned anomalies are generated. More importantly, we still do not know the consequences of mild and moderate alterations produced by an age-related oxidative stress in male germ cells. For instance, dysregulation of the transcription patterns of non-coding RNAs and its potential effects for the next generation have not been studied yet.

## 6 Biomaterial related ROS production in male infertility

In this section we present future perspectives in the regeneration of the male reproductive system, simultaneously pointing out ROS related research challenges.

### 6.1 Biomaterials and tissue engineering – the role of ROS in tissue reconstruction

The number of reported cancer cases, iatrogenic injuries, congenital abnormalities, and trauma of a male reproductive system has been increasing steadily over the last decade [140]. These disorders have negative impact on sperm production and its quality. As no other tissue shares similar characteristics with the male reproductive organ, none of the available surgical auto/allograft-based techniques are able to fully restore its functionality and preserve fertility.

Bioengineering brings novel solutions to genitourethral reconstruction and regeneration in male patients. So far autologous skin or osteocutaneous flaps have been combined with the synthetic systems such as silicone pumps [141,142]. These procedures are commonly applied in clinics, however do not satisfy needs of patients. Therefore, researchers are looking for a way to obtain functional corporal tissues, testicles or prostate using various materials and cell types [143,144]. The link between oxidative stress and implant failure has already been reported in the process of reconstruction of various organs [[145], [146], [147]]. Restoring male reproductive organs by using biomaterials is relatively new and future research should be directed based on the lessons learned elsewhere. Therefore, it is important to discuss the potential role of reactive oxygen species in a performance of genitourethral implants obtained by different engineering strategies.

Initially, non-degradable materials were of common interest for the male organ reconstruction, however their use resulted in many complications such as calcification, poor graft integration with surrounding tissues and ROS-related immune rejections [148]. Therefore, they were replaced by biodegradable polymers. The main reason of their application was that over the regeneration time they could be gradually replaced by native tissue. Synthetic polymers constitutes the largest subgroup of biodegradable materials in genitourethral regeneration and reconstruction due to their sufficient biocompatibility and ability to resist immunological rejection. In particular, poly-lactones such as poly-lactic acid (PLA), poly-glycolic acid (PGA), and poly-caprolactone (PCL), and their copolymers were considered to be the most promising biodegradable polymers, as they can be easily subjected to thermoplastic processing and possess the desired mechanical properties [149]. The poly (lactic-co-glycolic acid) (PLGA) scaffolds successfully seeded with normal human corporal smooth muscle cells (SMC) and human endothelial cells (EC) have formed vascularized erectile tissue (cavernosal muscle) after implantation into athymic mice. Nevertheless, the architecture and functionality of reconstructed tissue differed from the native one [150]. Researchers were not able to obtain implants that will provide sufficient level of the nitric oxide (NO*) production by cavernous endothelial cells for a penile erection. Moreover, the poly-lactone based biomaterial implants led to a three-fold increase in ROS production at surgery sites over a month [151]. It could be considered as an advantage for the reconstruction of various organs in a human body as ROS can trigger material degradation and substitution by native tissues. On the other hand in the case of the genitourethral grafts it may affect functionality of spermatozoa.

The natural polymers and acellular matrices could overcome these drawbacks. Kwon et al. have used the acellular matrices seeded with autologous corpus cavernosal smooth muscle cells and endothelial cells in order to replace a cross sectional segments of both of the corporal bodies in rabbits [152]. Unfortunately, researchers were not able to obtain implants with physiological intracaveronsal pressure due too low density of the smooth muscle cells repopulating implant. Chen et al. tested dynamic cell seeding using a bioreactor to overcome this limitation [153]. They have successfully recreated the corporal tissue by dynamic seeding of cavernosal collagen matrices with autologous smooth muscle and endothelial cells. Despite of tremendous progress in the field over the last few years, so far, the nerve structures have been omitted in engineering of the genitourethral implants. It is known that neural mediators such as NO* are crucial for a penile erection [154,155]. Increasing evidence indicates that NOS and NO* are associated with male infertility. However, there is not much known about how NO* that is produced by nerve structures located in the reproductive male organ influences sperm dynamics, morphology and acrosome reactions. Therefore, the next step in the engineering of reproductive male organ should be oriented towards nerve system reconstruction. In our opinion this will simultaneously bring a need for better understanding susceptibility of neural signalling to the oxidative stress.

Recently, researchers have also been working on the reconstitution of human testis niche using porous and nanofiber scaffolds obtained from natural polymers [156,157]. Moreover, Gohbara et al. were able to produce sperms in vitro using stem cells [158]. In the future, artificial niches produced by combination of scaffolds with stem cells might be sufficient to mimic and replace testicles [159]. In the previous paragraphs of this review, we have shown the crucial role of the ROS signalling in the spermatozoa maturation and physiological functions. This brings us to the conclusion that in order to progress in engineering of the functional implants of testicles we need to better understand and recreate redox conditions.

The results of the discussed studies bring us one step closer to finding clinical solutions. However, the relevance of oxidative stress triggered by the non-specific host response to implanted biomaterials remains to be elucidated. Moreover, it would be worth investigating if newly engineered implants can provide balanced ROS levels for physiological signalling.

### 6.2 Nanoparticles – ROS contribution in opportunities and risks

Recent advances in nanotechnology resulted in the development of various nanoparticle (NP) formulations with functionalities desired by researchers from many biomedical fields. Their distinctive properties make them also potent tools for assisted reproductive techniques. Herein, we would like to summarize opportunities and risks that application of nanoparticles brings into the field of male fertility and emphasize the link to the ROS production.

So far NPs have been successfully used as components of hybrid micromotors to restore sperm motility as well as for sperm separation and labelling [160]. Barchanski et al. have labelled sperms with bio-conjugated gold NPs [161,162]. Gamrad et al. reported successful binding of oligonucleotide functionalised gold nanoparticles to specific sites of the Y chromosome [163]. However, none of these researchers have investigated effect of gold probes on the ROS level in sperm cells. Vasquez et al. have developed bioluminescent magnetic nanoparticles modified with firefly luciferase as potential probes for imaging and tracking spermatozoa [164]. It has been suggested that nanoparticles conjugated with specific quality markers may help to investigate effects of the oxidative stress during semen storage at low temperatures [165]. Moreover, researchers have reported that magnetic beads enable efficient selection of the most vital spermatozoa within an ejaculate. The nanoparticles coated with molecules of high affinity to the particular cell biomarkers, such as annexin V or anti-ubiquitin antibodies, have been applied together with magnetic field to remove defective sperms from semen [166]. Feugang et al. have used the Fe_3_O_4_ NPs coated with lectins in order to select a subpopulation of highly motile spermatozoa [167]. It has been shown that the level of ROS was not different between control and sperms selected with nanoparticles. On the other hand, Wang at al. in their review have summarized effects of magnetic field on ROS generation, and found that in most cases the magnetic field was responsible for an increase of ROS level in multiple types of cells, including spermatozoa [168]. This discrepancy might be due to the magnetic field parameters rather than the presence or type of particles. Nevertheless, further investigations are crucial for a full understanding of the effects of sperm labelling and separation with magnetic particles on ROS production.

Beside sperm separation and labelling nanoparticles have been gaining interest as nanocarriers for improving transgenesis and targeted delivery of molecules. Makhluf et al. have designed magnetic NPs conjugated with an anti-kinase C antibody [169]. They have observed up-take of the nanocarriers and binding to the specific antigen in sperm cells. Similarly, it has been shown that mesoporous silica NPs loaded with either a fluorescent nucleic acid or a fluorescent protein are promising candidates for molecule delivery into male gametes [170]. Researcher have not found any adverse effect upon the main parameters of sperm functions, however they have not tested for changes in the ROS level yet.

Recently, NPs have also been applied in semen as antioxidants. Falachi et al. have determined the effects of storage of sperm cells with cerium oxide (CeO_2_) NPs at low temperatures [171]. Supplementation of cryopreserved semen with CeO_2,_ which is able to store oxygen and act as ROS scavenger, protected integrity of plasma membrane and DNA as well as improved sperm motility parameters. Nevertheless, observed effects could not be associated with the reduction of the intracellular ROS level. Authors of this research suggested that the sensitivity of commonly used H_2_DCFDA staining may not be sufficient to detect in sperm cells slight but impactful differences in ROS production. Selenium nanoparticles (SeNPs) are another type of the ROS scavengers used in several semen studies. While added to semen, the SeNPs improved its post-thawing quality [172]. Moreover, various animal studies have proved that diet supplementation with SeNPs resulted in an increase in antioxidant enzymes activity in semen [173]. Similar effects were observed after diet supplementation with zinc (ZnNPs) or zinc oxide (ZnONPs) nanoparticles [174,175]. Research of Rezvanfar et al. suggested that SeNPs also protects the quality of spermatozoa against oxidative stress triggered by the anticancer drugs [176].

On the other hand, some particles such as AgNPs, induce intracellular production of free radicals and those could be potentially used as antimicrobial agents. When added to seminal fluid they affect bacteria metabolism and decrease biofilm activity [177]. At the same time presence of AgNPs may have adverse effect on semen quality [178]. Sleiman et al. have presented that oral AgNPs administration resulted in significantly lower sperm production due to oxidative stress [179]. Similarly, zinc oxide and nickel nanoparticles (NiNPs) have been regarded as triggers of an excessive ROS production [180,181].

These findings should raise awareness of not only opportunities but also risks associated with daily application of nanoparticles. Notably, NPs are able to cross the blood–testis barriers. This is raising concerns about their potentially hazardous effects on male fertility. So far only little information is available about NPs induced oxidative stress in the reproductive system. Future research should lead to better understanding of the effects of NPs at cellular and tissue level.

## 7 Measuring ROS and sperm parameters

Multiple sperm parameters were already used for their assessment of the quality of spermatozoa for ART. According to the WHO guidelines, first macroscopic characteristics are measured. Here motility, vitality (membrane integrity) concentration and the total sperm count can be measured [182]. The most researched and understood parameters of sperm quality are concentration, motility and morphology. They are thought to have the best positive predictive value for ART outcomes, measured in pregnancies [183]. However, as our knowledge of sperm physiology and pathology increases, other parameters, like ROS levels, DNA integrity and lipid peroxidation, become potentially more important [184]. How these molecular factors are measured is described further below.

### 7.1 Detecting ROS directly

The most direct ways to determine ROS levels make use of chemicals that react with ROS and which can then be detected.

Chemiluminescence assays are among the most used techniques in the clinical practice [185,186]. This test relies on the use of luminol as probe for intracellular and extracellular ROS (H_2_O_2_ and superoxide) production [187]. For this purpose, whole ejaculates or neat semen are measured. The luminescence produced due to the oxidation of the luminol probe is measured with a luminometer and the signal is expressed as a relative light unit (RLU) per second per sperm concentration (RLU/s/10^6^ sperm). Agarwal et al. defined a cut off value to differentiate between infertile and healthy control donors. They found out that patients with reduced semen parameters (motility, morphology, concentration), targeted as infertile patients, present higher ROS levels than healthy control donors [188]. They further found a sensitivity and specificity of 76.4–93.8 and 53.3–68.8%, respectively. However, the quality of measurements depends on the selection of healthy control donors and their proven fertility capacity [188].

The oxidative stress produced by ROS can also be measured as static oxidation-reduction potential (sORP). This technique measures the transfer of electrons in the sample, taking into account both oxidising molecules and antioxidants at the same time. Higher amounts of sORP indicates higher oxidative stress which correlates with reduced semen parameters [189]. This assay shows a 60.4% sensitivity and 74.3% specificity for the detection of infertile patients in a multicenter study [190]. sORP seems to be a more stable assay over time and temperature for assessing oxidative stress than chemiluminescence methods and permits greater flexibility for sample handling [191]. However, this measurement can be influenced by the composition of the sample and the presence of polymorphonuclear leukocytes, in which case chemiluminescence gives a more accurate approximation for ROS levels [192].

Other types of assessment for ROS levels widely used in fundamental research are under still need to be evaluated to show if they have a predictive value on infertility before ART. These methods include fluorescent labels [193], which allows the identification of certain specific ROS molecules and their localisation in the sperm cells.

The most common probes are molecules that oxidise in the presence of ROS, producing a fluorescent molecule whose intensity can be later measured using flow cytometry or fluorescence microscopy. These labels can be bound to molecules that direct them to either cytosol or mitochondria. One of the most common labels for intracellular ROS in a wide variety of cells is dichlorodihydrofluorescein (H_2_DCFDA), which is used for the detection of hydrogen peroxide (H_2_O_2_), peroxynitrite (ONOO^-^) and hydroxyl radicals (OH*^-^). H_2_DCFDA detects mainly ROS generated close to the cell membrane in sperm cells. However, it seems that it could only detect an intense redox signal in around 3% of defective low‐density humans, which shows that is not suitable for diagnostic purposes [194,195]. On the other hand, dihydroethidium (DHE), commonly used to detect cytosolic superoxide (O2^•-^), appears to be capable of detecting the enhanced redox activity associated with defective, low‐density human spermatozoa [196,197].

Sperm mitochondrial ROS production can be assessed using MitoSox Red, a probe composed of two molecules: dihydroethidium (DHE), which can interact with superoxide radicals and triphenylphosphonium (TPP), which targets and directs DHE inside mitochondria. Amaral et al. applied this tool and found that sperm viability was shown to be negatively correlated with mitochondrial ROS production in sperms [197,198].

However, the application of fluorescent methods for ART requires a further understanding of the contribution of different types of ROS in the mechanisms towards infertility and further studies in population to define a cutoff value that could predict fertility status on patients.

### 7.2 Detecting damage caused by ROS

Since ROS are short lived and reactive it is usually difficult to measure them directly. Thus, measuring the damage they cause or the response of the cells to their presence instead are practical alternatives. As discussed earlier, damage can be done in principle to virtually any biomolecule. Damage usually leads to oxidation of these biomolecules. These oxidized versions of the molecules can then be detected with different analytical techniques. The most important.

methods that are currently used to assess ROS production in clinical samples are summarized in Table 1.

**Table 1:** Examples for some important techniques for detecting damage caused by ROS.

Technique	Method	Advantages	Disadvantages
2,4dinitrophenylhydrazine (DNPH) [199]	Detection of Protein carbonyl	Accessible and inexpensive	Laborious and time-consuming because it requires protein precipitation
High-performance liquid chromatography (HPLC) [200]	Detection of lipids, separate, identify, and quantify each component in a mixture	Speed, high sensitivity and specify	Cost, and complexity
Gas chromatography-mass spectrometry (GC-MS) [201]	Separate chemical mixtures and identifies the components at a molecular level	High specificity, selectivity, sensitivity, identify a wide range of DNA base products	Limited to thermally stable and volatile compounds
Enzyme-linked immunosorbent assay (ELISA) [202,203]	DNA damage marker, based on antigen–antibody reaction	Easy, high specificity and sensitivity	Price, inaccuracy, and insufficient blocking of immobilized antigen
Capillary zone electrophoresis (CZE) [204]	Separation of charged molecules (DNA, RNA, proteins) and transport them by an electrical field	High separation efficiency, speed, low sample consumption, low waste, ease	Relatively poor accuracy
Quantitative polymerase chain reaction [205]	Evaluating the relative gene expression of oxidative stress genes	Sensitivity, ability to compare damage to nuclear (nDNA) and to mitochondrial (mtDNA) from the same sample	Susceptible to inhibitors present in some biological samples
RNA sequencing [206,207]	Quantify and sequence of RNA in a sample, analyse genes expression	High dynamic range, not reliant on previous sequence information, high accuracy	Requires high power computing facilities, cost, analysis can be complex.

### 7.3 DNA-fragmentation

A recent meta-analysis found a link between high frequency sperm DNA fragmentation and recurrent pregnancy loss. Here, studies were included that could produce an explicit mean/median for DNA fragmentation, with acceptable confidence intervals or ranges [28]. For more explicit evidence and better interpretation, it remains beneficial to seek more controlled and standardized methods.

Contrarily, another meta-analysis on the predictive value of sperm DNA fragmentation on the outcome of ART found limited evidence due to its large amount of heterogeneity [27].

Still, measuring DNA fragmentation, hereby linking it to levels of ROS, could be a valuable tool in the development and success of ART. When interpreting these results, we must bear in mind that no clear cut-off score has been defined for sperm DNA fragmentation. Many ways exist to measure DNA fragmentation, as explained below, and ART outcomes. If clinical implementation is the goal, RCT's must be done using standardized scores, measures and equal outcomes.

Currently DNA damage in spermatozoa, for use of ART, is measured in the following ways: Comet assay; sperm chromatin structure assay (SCSA); sperm chromatin dispersion (SCD); TUNEL.

Firstly, the Comet assay, using decompaction and electrophoresis to look at individual spermatozoa, is performed under alkaline or neutral conditions and can show single and double stranded DNA fragmentations. Secondly, the SCSA and SCD test rely on the denaturing capacity of the sperm chromatin [208]. The SCSA bases its results on the DNA fragmentation index, which is the percentage in the sample that has measurable increased red fluorescence. The red fluorescence is due to acridine orange (dye) attaching to a single strand portion of DNA at sites of DNA strand breaks. It then collapses into a crystal, which exposed to blue light, produces a metachromatic shift to red fluorescence [27]. The SCD shows nuclear dispersion in the form of a halo (dispersed DNA loops after removal of nuclear proteins, detected in fluorescence microscopy [209]). It differentiates non-fragmented spermatozoa with halo, from fragmented spermatozoa without the halo. Lastly, the TUNEL assay labels the 3’ free ends of the DNA by using a terminal TdT transferase. It depicts more labelling on spermatozoa with more DNA fragmentation.

Although many (usually time-consuming) methods exist to measure DNA fragmentation, no standardization of the processes exist. To many scientists’ frustration, a lot of research has been done, yet no clear cut-off values have come forward. Comparing methods without clear cut-off scores or pre-determined outcomes makes it hard to interpret and improve results. Additional issues arrise from the fact that DNA fragmentation is measured from large ensemble of cells including non-vital spermatozoa. Thus, it does not reflect the state of the healthy cells correctly. Besides ROS, other intrinsic factors such as deficiencies in recombination and abnormal maturation cause DNA fragmentation. In addition, external factors such as age, abstinence, temperature (of testicles), varicocele and the effects of clinical procedures (eg. chemo- and radiotherapy) can negatively influence DNA integrity [210].

Nevertheless, the relationship between ROS and DNA integrity could be highlighted by linking methods to measure both. When it comes to their correlation, one test might show more specificity and sensitivity to ROS and its DNA damage than another.

## 8 Conclusions and outlook

Studies show relations of lifestyle and male fertility changes due to oxidative stress. However, it remains a challenge how we can best popularize this scientific information to change daily life habits and therefore decrease the number of infertility cases.

ROS is undeniably an important factor for both healthy sperm development and function as well as processes which reduce fertility. Several factors have been identified which link ROS production and male subfertility or infertility. However, there is still very limited knowledge available about how exactly they are linked or to what extent they matter. This is the case on the cell level but even more when assessing the relevance of clinical interventions. Better understanding of the effect of lifestyle habits and their association of male subfertility will need to be acquired. There are also many seemingly conflicting results which emphasizes the need to standardise how and where we measure ROS and how exactly we determine success of an intervention. The fact that many factors have impact on ROS production and subfertility also strongly contributes to this problem.

Further, it is important for ART to control ROS production for any treatment that the ejaculate undergoes to avoid additional damage caused by the very methods that were meant to improve sperm quality or conserve sperm.

Another point that would significantly advance the field is the possibility to differentiate between different ROS. Here a new technology which identifies radicals specifically is promising.

## Declaration of competing interest

The authors have no conflict of interest to declare.

## Footnotes

1 MET definition: ratio of the rate of energy expended during an activity to the rate of energy expended at rest. Multiplying the time spent in each activity by the MET value corresponds to that activity, MET/h. 20–40 METs-h/week refers to moderate physical activities, breathing somewhat harder than normal. 40–80 METs-h/week refers to hard physical activities, breathing much harder than normal. >80 METs-h/week refers to exhausting physical activities.
